# The Effect of Capsulotomy Shape on Intraocular Light-Scattering after Nd:YAG Laser Capsulotomy

**DOI:** 10.1155/2020/4153109

**Published:** 2020-03-23

**Authors:** Jun Li, Zhe Yu, Hui Song

**Affiliations:** Tianjin Eye Hospital, Tianjin Key Lab of Ophthalmology and Visual Science, Tianjin Eye Institute, Clinical College of Ophthalmology, Tianjin Medical University, Nankai University Eye Hospital, Tianjin, China

## Abstract

**Purpose:**

To investigate the effects of capsulotomy shape on the visual acuity and visual quality after neodymium: yttrium aluminum garnet laser capsulotomy.

**Methods:**

In this study, a total of 42 eyes from 35 patients with posterior capsule opacification were divided into the circular and cruciate groups. The corrected distance visual acuity (CDVA), objective scatter index (OSI), modulation transfer function cutoff (MTF cutoff), Strehl ratio, and Optical Quality Analysis System values at contrasts of 100%, 20%, and 9% (OV-100, OV-20, and OV-9) were measured at precapsulotomy and 1 week and 1 month postcapsulotomy. The pseudophakic dysphotopsia questionnaire (PDQ) was used to evaluate the subjects' satisfaction with treatment.

**Results:**

OSI values were significantly higher in the cruciate group than in the circular group at 1 week and 1 month after capsulotomy (*P*=0.013 and *P* < 0.001). No significant difference was found in the OSI values between the two groups before capsulotomy (*t* = 0.52; *P*=0.61). The decrease in OSI was higher in the circular group than in the cruciate group at 1 week and 1 month after capsulotomy (*P*=0.036 and *P*=0.019). No significant differences were found in the Strehl ratio, MTF cutoff, CDVA, OV-100, OV-20, and OV-9 between the two groups at 1 week and 1 month after capsulotomy (*P* > 0.05). The PDQ results showed that patients with circular-shaped capsulotomy complained less with intolerance of bright lights than those with cruciate-shaped capsulotomy.

**Conclusions:**

Circular-shaped capsulotomy can induce less intraocular light scattering and increase patient satisfaction.

## 1. Introduction

Phacoemulsification combined with foldable intraocular lens (IOLs) implantation can markedly improve visual acuity and contrast sensitivity in patients with cataracts. However, posterior capsular opacification (PCO) is a common complication after cataract surgery. Lundqvist and Mönestam found that over one-third of patients received neodymium: yttrium aluminum garnet (Nd:YAG) laser capsulotomy for PCO in 10 years after cataract surgery [1]. Schaumberg et al. [2] showed that the incidences of PCO at 1, 3, and 5 years after cataract surgery are 11.8%, 20.7%, and 28.4%, respectively. Ursell et al. [3] followed up 13,329 eyes implanted with AcrySof IOL, 19,025 eyes implanted with non-AcrySof hydrophobic IOL, and 19,808 eyes implanted with non-AcrySof hydrophilic IOL for 3 years and found a 3-year incidence of PCO and Nd:YAG capsulotomy ranging from 4.7% to 14.8% according to the different IOL materials used. Ambroz et al. [4] found PCO in 30.9% of the surveyed eyes at 18.4–50.2 months after pediatric cataract surgery. In pediatric cataract patients without posterior capsulotomy and anterior vitrectomy, the incidence of PCO was as high as 70% [5].

PCO can markedly degrade visual function, including visual acuity and contrast sensitivity [6, 7]. Most patients with PCO suffer from disability glare, which reduces retinal image contrast [8, 9]. Nd:YAG capsulotomy effectively improves the visual acuity, contrast sensitivity, and glare sensitivity of patients with PCO. It can also decrease intraocular light scattering and improve patient satisfaction following treatment [10, 11].

**Figure 1:**
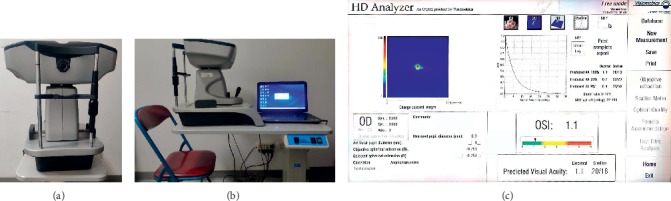
Front (a) and lateral view (b) of the optical quality analysis system, and the printout report (c).

Intraocular light scattering is an important parameter used to evaluate visual function [12]. It can be perceived as glare, halos, blinding at night while driving, and hazy vision. Montenegro et al. [10] found that increased intraocular light scattering can cause veiling luminance on the retina, which leads to glare, halos, and blinding at night. The condition may severely degrade visual performance and retinal image quality and is an important cause of visual function impairment in pseudophakic eyes. The parameters measured by the Optical Quality Analysis SystemII (OQAS, Visiometrics S.L., Terrasa, Spain) (Figure 1), such as modulation transfer function (MTF) cutoff, Strehl ratio, objective scatter index (OSI), and OQAS values at contrasts of 100% (OV-100), 20% (OV-20), and 9% (OV-9), are widely used to evaluate the intraocular light scattering and objective optical quality of eyes in clinics. MTF is the ratio of contrast between the retinal image and the original scene, and the MTF cutoff is defined as the cutoff frequency at 1% of the maximum MTF [13]. The Strehl ratio is the ratio of the central intensity of the point image between the measured and ideal eye. OV-100, OV-20, and OV-9 are the OQAS values calculated by the OQAS system at contrasts of 100%, 20%, and 9%, respectively. OV-100 is equal to the MTF cutoff frequency divided by 30 cycles per degree (cpd), whereas OV-20 and OV-9 are 0.05 and 0.01 of the MTF, respectively. OSI is the ratio of light intensity between the peripheral annular zone (12 min of arc) and the central peak zone (within 1 min of arc). A higher OSI value indicates more intraocular light scattering. Higher values of MTF cutoff, Strehl ratio, OV-100, OV-20, and OV-9 indicate better visual quality. These parameters can help evaluate the visual quality of the human eye objectively [13, 14].

Nd:YAG laser capsulotomy size can affect visual function [15]. Holladay et al. [16] found that a smaller capsulotomy opening increases light scattering. However, to date, whether the shape of Nd:YAG laser capsulotomy affects visual function and the effects of Nd:YAG laser capsulotomy shape on visual acuity, intraocular light scattering, and MTF remain to be evaluated. In this study, Nd:YAG laser capsulotomy with a circular or cruciate shape was performed to evaluate the effects of shape on visual acuity, intraocular light scattering, and MTF.

## 2. Materials and Methods

### 2.1. Subjects

The study protocol was approved by the local ethics committee. Informed consent was obtained from the participants, and all procedures followed the tenets of the Declaration of Helsinki. A total of 42 eyes of 35 patients with PCO were involved in this study between July 2017 and December 2018. No phacoemulsification complications were found in all patients. Ophthalmological examinations, including corrected distance visual acuity (CDVA), refractive measurements, intraocular pressure (IOP), and slit-lamp and fundus examination, were performed before and after Nd:YAG laser capsulotomy. The patients were assigned to circular and cruciate capsulotomy groups sequentially.

The inclusion criteria were as follows: age between 50 years and 90 years, postoperative astigmatism of less than 1.00 diopter (D), same implanted IOL (Akreos Adapt, Bausch & Lomb, USA), IOL power between +18.00 D and +24.00 D, and uncomplicated surgery with well-centered IOL in the capsular bag. Patients with ocular pathologies (e.g., high myopic, corneal opacities, retinopathy, maculopathy, and glaucoma), systemic diseases, serious anterior chamber inflammation after capsulotomy, and a history of ocular surgery were excluded. During capsulotomy, the laser might hit the optic of the IOL, which could result in spots on the optic. The spots may induce extra intraocular light scattering. Thus, the patients with spots on the optic of the IOL were also excluded.

**Figure 2:**
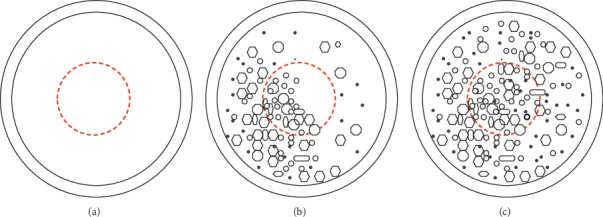
Subjective grading of PCO. (a) “0 = a completely clear capsule,” (b) “5 = half of the capsule covered with PCO,” and (c) “10 = a capsule completely covered with PCO.” The red dotted circle is the 4.0 mm central zone of the posterior capsule.

All patients reported blurred vision. Subjective grading was used in this study. PCO was subjectively graded on a scale of 0 to 10 by three doctors, and the average was recorded. A posterior capsule completely covered with severe PCO received a score of 10, whereas a completely clear posterior capsule received a score of 0. The mean PCO score was recorded. The central 4.0 mm zone of the posterior capsule was the key area observed (Figure 2). Findl et al. [17] reported that the subjective grading correlated well with the objective Automated Quantification of After-Cataract (AQUA) system and the Evaluation of Posterior Capsular Opacification (EPCO) system. The AQUA system can automatically analyze the retroillumination images of PCO within the capsulorhexis region and calculate a score between 0 and 10 (0 = a clear capsule; 10 = a capsule completely covered with PCO) [11, 18]. The EPCO system can also evaluate the photography of PCO, and the EPCO score is calculated by a computer (0 = a clear capsule; 4 = a capsule with severe PCO) [19]. Findl et al. [17] found that the subjective grading by examiners and objective analytic systems (such as AQUA and EPCO system) showed good reproducibility and correlated well with each other.

### 2.2. Nd:YAG Laser Capsulotomy Technique

Before capsulotomy, the pupils were dilated with topical 0.5% tropicamide and 0.5% phenylephrine eye drops (Mydrin-P, Santen Pharmaceutical, Osaka, Japan). Then, 0.5% proparacaine hydrochloride (Alcaine, Alcon Co., USA) was used for topical anesthesia. A contact lens was applied to facilitate accurate focusing after topical anesthesia. All Nd:YAG laser capsulotomy procedures were performed by the same surgeon (J.L.) using an Nd:YAG laser (Ellex Inc., Adelaide, AUS). A 4-mm light band of slip lamp was used to examine the capsulotomy area at 0°, 45°, 90°, and 135° in all patients. Thus, we ensured that the diameter of capsulotomy was 4.0 mm at the specified degrees. The residual larger fragments in the capsulotomy area were also cleared by the Nd:YAG laser in the cruciate group. After capsulotomy, 1% fluorometholone (Santen Pharmaceutical, Osaka, Japan) four times daily for 3 days was prescribed.

**Figure 3:**
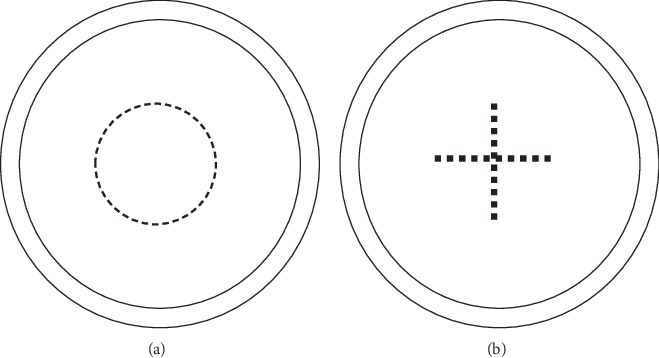
Circular- (a) and cruciate-shaped (b) YAG laser capsulotomies.

Patients were divided into two groups according to the shape of capsulotomy. Cruciate-shaped capsulotomy was performed in 21 eyes (cruciate group), whereas circular-shaped capsulotomy was performed in another 21 eyes (circular group). The diameter of capsulotomy was 4.0 mm, which was measured by a slit-beam ruler. The laser energy was set to 2.0–2.5 mJ in the cruciate group and 1.5–2.0 mJ in the circular group. In the circular group, we decreased the Nd:YAG laser energy to obtain a round, centered, and relatively smooth foramen. Once the central posterior capsule was cut off by a low-energy laser, we increased the laser energy (from 2.5 mJ/pulse to 3.5 mJ/pulse) and smashed the central posterior capsule. In the cruciate group, we performed cruciate-shaped capsulotomy using laser energy levels ranging from 2.0 mJ/pulse to 2.5 mJ/pulse. The capsular debris in the optical axis was all cleared (Figure 3).

### 2.3. Ophthalmologic Measurements

At before capsulotomy and 1 week and 1 month after capsulotomy, the CDVA, slit-lamp, and fundus examinations were performed. The OSI, MTF cutoff, Strehl ratio, OV-100, OV-20, and OV-9 were measured using OQAS II (Visiometrics S.L., Terrasa, Spain). The spherical refractive error was automatically corrected internally by OQAS (from −3D to +3D). The spherical refractive error of more than ±3D and astigmatism were corrected by placing an appropriate spherical and cylindrical lens in front of the eye. The pupils were fully dilated with topical 0.5% tropicamide and 0.5% phenylephrine eye drops (Mydrin-P, Santen Pharmaceutical, Osaka, Japan) before examination. The parameters were automatically measured by OQAS under a 4.0-mm artificial pupil, which was controlled by a diaphragm wheel inside the OQAS. All subjects were measured three times, and the average was recorded.

The pseudophakic dysphotopsia questionnaire (PDQ) designed by Dr. Olson R.J. was used to evaluate the patients' satisfaction at 1 week and 1 month postcapsulotomy. The PDQ includes nine questions to rate the satisfaction with different pseudophakic dysphotopsia symptoms. For each question, the subject was asked to rate their satisfaction with dysphotopsia symptoms from 0 (no problem) to 10 (debilitating). The nine questions include satisfaction with bright light, oncoming headlights at night, halos, glares, flashes of light, dark or shadows at the side of vision, flickering shadow around lights, and semicircular shadow. And an additional question is the overall satisfaction with the vision (0 = totally unsatisfied; 10 = totally satisfied). The PDQ is considered to evaluate the satisfaction of pseudophakic dysphotopsias accurately and has good reproducibility [20].

### 2.4. Statistical Analysis

Data were statistically analyzed using SPSS computer package 17.0 (SPSS Inc., Chicago, Ill, USA). The normality of data distribution was confirmed with the Shapiro–Wilk test. The decimal Snellen visual acuity was converted into a logMAR scale. The independent sample *t*-test for parametric variables and the Mann–Whitley *U* test for nonparametric variables were used to compare the means between the two groups.

## 3. Results

Forty-two eyes of 35 patients (10 males and 25 females) were examined. All patients completed the 1-month follow-up. The mean age was 68.55 ± 10.63 years (range, 51–87 years). No significant differences in age and gender were observed between the two groups (*P*=0.246 and *P*=0.747, respectively). No associated complications (e.g., serious anterior chamber inflammation, macular edema, anterior hyaloid damage, and retinal detachment) were observed in all patients.  Table 1 shows the OSI, spherical equivalent (SE), and CDVA at pre- and postcapsulotomy.

The applied laser energies were 93.48 ± 31.04 mJ in the circular group and 77.19 ± 22.47 mJ in the cruciate group. No significant difference was observed in the applied laser energy between the two groups (*t* = 1.948, *P*=0.059). The SEs at 1 week postcapsulotomy were −0.25 ± 0.68 D (range −1.25 D to 1.25 D) in the circular group and −0.11 ± 0.83 D (range −1.25 D to 1.25 D) in the cruciate group. The SEs at 1 month postcapsulotomy were −0.30 ± 0.63 D (range −1.75 D to 1.75 D) in the circular group and −0.12 ± 0.74 D (range −1.75 D to 1.25 D) in the cruciate group. No significant difference was observed between the two groups in the SE at 1 week and 1 month postcapsulotomy (*t* = −0.608, *P*=0.547; and *t* = −0.839, *P*=0.407).

The medians of CDVA at 1 week postcapsulotomy were 0.10 logMAR (range 0 to 0.14 logMAR) in the circular group and 0.10 logMAR (range 0 to 0.10 logMAR) in the cruciate group. No significant difference was found between the two groups in the CDVA at 1 week postcapsulotomy (*U* = 207.500; *P*=0.724). The medians of the CDVA at 1 month postcapsulotomy were (range 0 to 0.10 logMAR) in the circular group and 0.00 logMAR (range 0 to 0.10 logMAR) in the cruciate group. No significant difference was found in the CDVA at 1 month postcapsulotomy between the two groups (*U* = 180.00; *P*=0.250).

**Figure 4:**
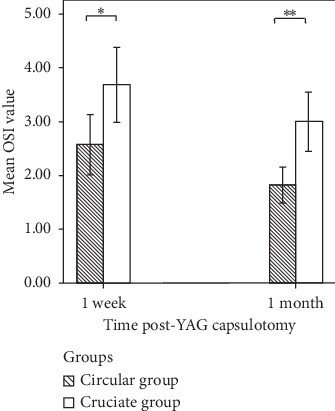
OSI values were significantly higher in the cruciate group than in the circular group at 1 week and 1 month postcapsulotomy.

**Figure 5:**
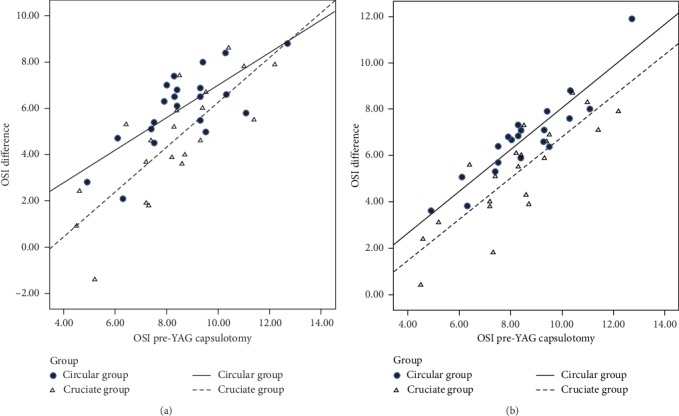
The decrease in OSI was higher in the circular group than in the cruciate group at 1 week (a) and 1 month (b) postcapsulotomy.

The mean OSI values at 1 week postcapsulotomy were 2.57 ± 1.23 in the circular group and 3.69 ± 1.53 in the cruciate group. The OSI was higher in the cruciate group than in the circular group (*t* = −2.606; *P*=0.013). The mean OSI values at 1 month postcapsulotomy were 1.82 ± 0.73 in the circular group and 3.00 ± 1.21 in the cruciate group. The OSI was significantly higher in the cruciate group than in the circular group (*t* = −3.823; *P* < 0.001) (Figure 4). The decrease in OSI compared with preoperative OSI was significantly higher in the circular group than in the cruciate group at 1 week and 1 month postcapsulotomy (*t* = 2.164, *P*=0.036; and *t* = 1.582, *P*=0.019) (Figure 5).

**Figure 6:**
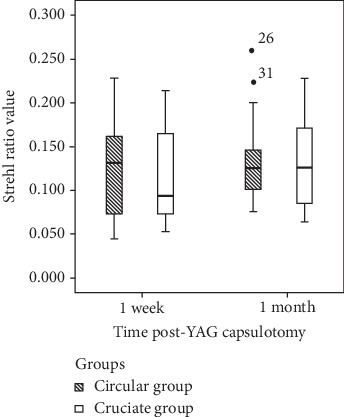
No significant difference was found in the Strehl ratio between the circular and cruciate groups at 1 week and 1 month post-YAG laser capsulotomy.

No significant difference was found in the Strehl ratio between the circular and cruciate groups at 1 week postcapsulotomy (0.132 (0.067–0.162) VS 0.094 (0.072–0.180), *U* = 217.000; *P*=0.930). Similarly, no significant difference was found in the Strehl ratio between the circular and cruciate groups at 1 month postcapsulotomy (0.126 (0.101–0.148) VS 0.126 (0.083–0.173), *U* = 206.000; *P*=0.715) (Figure 6 and Table 2).

**Figure 7:**
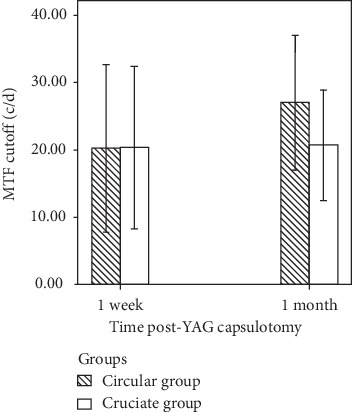
No significant difference was found in the MTF cutoff between the circular and cruciate groups at 1 week and 1 month post-YAG laser capsulotomy.

The mean MTF cutoff values at 1 week postcapsulotomy were 20.242 ± 12.407 cpd in the circular group and 20.352 ± 12.055 cpd in the cruciate group. No significant difference was found in the MTF cutoff between the two groups at 1 week postcapsulotomy (*t* = 0.015; *P*=0.988). Similarly, no significant difference was found in the MTF cutoff value between the circular and cruciate groups 1 month postcapsulotomy (27.013 ± 10.029 cpd VS 21.628 ± 7.693 cpd; *t* = 1.952; *P*=0.058) (Figure 7 and Table 2).

No significant differences were found in the OV-100, OV-20, and OV-9 between the circular and cruciate groups at 1 week postcapsulotomy (0.600 (0.250–0.900) VS 0.600 (0.300–1.150), *U* = 204.000, *P*=0.677; 0.400 (0.200–0.600) VS 0.300 (0.200–0.800), *U* = 15.500, *P*=0.898; 0.200 (0.100–0.400) VS 0.200 (0.100–0.400), *U* = 202.500, *P*=0.643). Similarly, no significant difference was found in the OV-100, OV-20, OV-9 between the circular and cruciate groups 1 month postcapsulotomy (0.800 (0.600–1.200) VS 0.800 (0.400–0.900), *U* = 156.5, *P*=0.105; 0.600 (0.400–0.850) VS 0.500 (0.300–0.600), *U* = 145.500, *P*=0.056; 0.400 (0.200–0.400) VS 0.300 (0.200–0.400), *U* = 183.500, *P*=0.336) (Table 2).

At 1 week postcapsulotomy, the PDQ results showed that 16 (76.19%) of 21 patients in the circular group and 21 (100%) of 21 patients in the cruciate group had 1 or more complaints with intolerance of bright lights. Eight patients (38.10%) in the circular group and twelve patients (57.14%) in the cruciate group had complaints rated as 5 or more (with 0 being no complaint and 10 being debilitating). One patient (4.76%) in the circular group and two patients (9.52%) in the cruciate group had complaints rated as 8 or more. One month after laser capsulotomy, the PDQ results showed that 14 (66.67%) of 21 patients in the circular group and 19 (90.48%) of 21 patients in the cruciate group had 1 or more complaints with intolerance of bright lights. One patient (4.76%) in the circular group and 7 patients (33.33%) in the cruciate group had complaints rated as 5 or more (with 0 being no complaint and 10 being debilitating) (Table 3). No patient in the two groups had complaints rated as 8 or more. The scores for the overall satisfaction of surgery (0 = totally unsatisfied; 10 = totally satisfied) were significantly higher in the circular group than in the cruciate group at 1 week and 1 month postcapsulotomy (*U* = 137.000, *P*=0.035; and *U* = 140.000, *P*=0.041) (Table 3).

## 4. Discussion

Nd:YAG laser capsulotomy can markedly improve visual acuity in patients with PCO [21]. However, visual acuity is only one component of visual function. Intraocular light scattering causes patient dissatisfaction after intraocular surgery. In this study, we evaluated the effects of capsulotomy shape on visual function after capsulotomy. The mean OSI values of the cruciate group at 1 week and 1 month postcapsulotomy were markedly higher than those of the circular group. However, the intraocular light scattering might also have been induced by the PCO before capsulotomy [11]. The OSI values of the two groups before capsulotomy were also measured, and no significant difference was found. In addition, the decrease in OSI compared with the preoperative OSI was higher in the circular group than in the cruciate group at 1 week and 1 month postcapsulotomy. Thus, we inferred that circular-shaped capsulotomy induced less intraocular light scattering. As OSI is only an objective light-scattering parameter, the patients' subjective sense should also be evaluated. The pseudophakic dysphotopsia survey designed by Dr. Olson R.J. was used in the present study [20], and our results showed that the scores of overall satisfaction were higher in the circular group than in the cruciate group at 1 week and 1 month postcapsulotomy. Patients with circular-shaped capsulotomy also complained less of intolerance of bright lights than those with cruciate-shaped capsulotomy. Circular-shaped capsulotomy induced less intraocular light scattering than cruciate-shaped capsulotomy. Capsulotomy size affects intraocular light scattering. Goble et al. [22] reported that patients who received wide capsulotomies show less forward light scattering than those who received narrow treatment. Montenegro et al. [10] reported that small capsulotomies could increase intraocular straylight. In this study, the capsulotomy diameters were of the same size (4.0 mm) in the two groups. We inferred that capsulotomy shape can also affect intraocular light scattering. Capsule remnants are important factors that induce light scattering and glare disability [22]. Montenegro et al. [10] found that the percentage of the pupil area with capsule remnants considerably contributed to intraocular light scattering after Nd:YAG laser capsulotomy. Nd:YAG laser capsulotomy with a circular shape and lower energy results in a smoother capsulotomy opening edge and fewer capsule remnants in the pupil areas; by contrast, cruciate-shaped capsulotomy with higher energy often produces more capsule remnants and jagged edges, which can increase light scattering.

Our results show that the CDVA did not significantly differ between the two groups. Intraocular light scattering and best-corrected visual acuity (BCVA) have been reported to significantly improve after laser capsulotomy, and the former was shown to be independent of the latter. Before capsulotomy, the intraocular light scattering was moderately correlated with BCVA. After capsulotomy, no significant correlation was found between BCVA and intraocular light scattering [9]. Visual acuity and intraocular light scattering are entirely different descriptors of visual quality. Visual acuity encompasses the central 0.02° of the point spread function [23]. OSI measures the forward light scattering in a visual angle within 20 min of an arc by the double-pass method [24]. Visual acuity correlates well with contrast sensitivity, whereas visual acuity and contrast sensitivity are not well correlated with intraocular light scattering [12]. Van den Berg's [25] study on donor eye lenses also showed that light scattering does not affect the point spread function center. Visual acuity is weakly correlated with intraocular light scattering. Intraocular light scattering in the eye is spread through a large angle.

In the present study, a significant difference was found in the intraocular light scattering between the two groups, but no significant differences were found in the MTF cutoff, Strehl ratio, OV-100, OV-20, and OV-9 between the two groups at 1 week and 1 month after YAG laser capsulotomy. Pennos et al. [26] reported a strong correlation between the C-Quant log (s) and contrast sensitivity. C-Quant measures forward light scattering over a visual angle of 5°–10° by the compensation comparison method, whereas OQAS measures forward light scattering over a smaller visual angle within 20 min of an arc by the double-pass method [24]. In our study, OQAS was used to measure intraocular light scattering. Hence, the visual angle measured by C-Quant was larger than that measured by OQAS. Different visual angles may lead to variations between these measurements. The C-Quant straylight meter uses the compensation comparison method. During examination, the subject test field is divided into half fields, and the subject chooses the half field that flickers more intensely [27]. Hence, the examination results can be affected by the subjects' responses. The subject's age and education can also affect the examination results. The OQAS is based on the analysis of the double-pass image of a point source projected on the retina and evaluates forward light scattering. OQAS is an objective examination, and all procedures can be completed within a few minutes [14, 24]. Moreover, the repeatability of the OQAS measurements is slightly better than that of the C-Quant measurements. OQAS measurements provide a slightly higher intraclass correlation coefficient than C-Quant measurements [24].

## 5. Conclusion

In conclusion, circular-shaped capsulotomy induces less intraocular light scattering than cruciate-shaped capsulotomy. It does not require high laser energy. Considering these characteristics, circular-shaped capsulotomy can provide better satisfaction than cruciate-shaped capsulotomy.

## Figures and Tables

**Table 1:** The OSI, SE, and CDVA before and after capsulotomy.

	Circular group	Cruciate group	U or t	*P*
PCO score	7.30 ± 1.27	7.57 ± 1.14	−0.73	0.47
Laser energy	93.48 ± 31.04 mJ	77.19 ± 22.47 mJ	1.95	0.06
CDVA before capsulotomy	0.86 ± 0.40 logMAR	0.78 ± 0.43 logMAR	−0.78	0.44
CDVA 1 week after capsulotomy	0.09 ± 0.07 logMAR	0.08 ± 0.07 logMAR	207.50	0.72
CDVA 1 month after capsulotomy	0.06 ± 0.06 logMAR	0.04 ± 0.05 logMAR	180.00	0.25
OSI before capsulotomy	8.58 ± 1.76	8.27 ± 2.07	0.52	0.61
OSI 1 week after capsulotomy	2.57 ± 1.23	3.69 ± 1.53	−2.61	0.01
OSI 1 month after capsulotomy	1.82 ± 0.73	3.00 ± 1.21	3.82	<0.001
SE before capsulotomy	−0.67 ± 3.40 D	0.07 ± 3.50 D	−0.70	0.49
SE 1 week after capsulotomy	−0.25 ± 0.68 D	−0.11 ± 0.83 D	−0.61	0.55
SE 1 month after capsulotomy	−0.30 ± 0.63 D	−0.12 ± 0.74 D	−0.84	0.41

mJ = millijoules; OSI = objective scatter index; SE = spherical equivalent; CDVA = corrected distance visual acuity. The results are presented as mean ± SD.

**Table 2:** The Strehl ratio, MTF cutoff, OV-100, OV-20, and OV-9 of circular and cruciate groups at 1 week and 1 month after capsulotomy.

	Circular group	Cruciate group	*U* or *t*	*P*
Strehl ratio 1 week after capsulotomy	0.12 ± 0.06	0.12 ± 0.06	217.00	0.93
Strehl ratio 1 month after capsulotomy	0.14 ± 0.05	0.13 ± 0.05	206.00	0.72
MTF cutoff 1 week after capsulotomy	20.24 ± 12.41	20.35 ± 12.06	0.02	0.99
MTF cutoff 1 month after capsulotomy	27.01 ± 10.03	20.72 ± 8.19	1.95	0.06
OV-100 1 week after capsulotomy	0.65 ± 0.40	0.70 ± 0.41	204.00	0.68
OV-100 1 month after capsulotomy	0.95 ± 0.34	0.70 ± 0.28	156.50	0.11
OV-20 1 week after capsulotomy	0.46 ± 0.28	0.47 ± 0.30	15.50	0.90
OV-20 1 month after capsulotomy	0.63 ± 0.23	0.49 ± 0.21	145.50	0.06
OV-9 1 week after capsulotomy	0.25 ± 0.17	0.28 ± 0.17	202.50	0.64
OV-9 1 month after capsulotomy	0.34 ± 0.16	0.30 ± 0.16	183.50	0.34

MTF cutoff = modulation transfer function cutoff; OV-100 = Optical Quality Analysis System values at contrasts of 100%; OV-20 = Optical Quality Analysis System values at contrasts of 20%; OV-9 = Optical Quality Analysis System values at contrasts of 9%. The results were presented as mean ± SD.

**Table 3:** Results of the pseudophakic dysphotopsia questionnaire (PDQ).

PDQ question	Circular group						Cruciate group					
	1 week after YAG laser			1 month after YAG laser			1 week after YAG laser			1 month after YAG laser		
	Median (interquartile range)	Number (%)		Median (interquartile range)	Number (%)		Median (interquartile range)	Number (%)		Median (interquartile range)	Number (%)	
		With a score of ≥5	With a score of ≥8		With a score of ≥5	With a score of ≥8		With a score of ≥5	With a score of ≥8		With a score of ≥5	With a score of ≥8
1	2 (0–3)	4 (19.05)	0 (0)	0 (0–1.5)	0 (0)	0 (0)	2 (1–5)	6 (28.57)	0 (0)	0 (0–3)	3 (14.29)	0 (0)
2	1 (0–3)	4 (19.05)	0 (0)	0 (0–1.5)	0 (0)	0 (0)	1 (0–4.5)	5 (23.81)	1 (4.76)	1 (0–3)	2 (9.52)	0 (0)
3	0 (0–0)	1 (4.76)	1 (4.76)	0 (0–0)	0 (0)	0 (0)	0 (0–1.5)	2 (9.52)	2 (9.52)	0 (0–0)	2 (9.52)	0 (0)
4	0 (0–0)	0 (0)	0 (0)	0 (0–0)	0 (0)	0 (0)	0 (0–0.5)	0 (0)	0 (0)	0 (0–0)	0 (0)	0 (0)
5	0 (0–0)	1 (4.76)	0 (0)	0 (0–0)	1 (4.76)	0 (0)	0 (0–0)	1 (4.76)	0 (0)	0 (0–0)	0 (0)	0 (0)
6	0 (0–0)	1 (4.76)	1 (4.76)	0 (0–0)	0 (0)	0 (0)	0 (0–1.5)	3 (14.29)	2 (9.52)	0 (0–0.5)	2 (9.52)	0 (0)
7	0 (0–0.5)	1 (4.76)	0 (0)	0 (0–0)	0 (0)	0 (0)	0 (0–1.5)	1 (4.76)	0 (0)	0 (0–0.5)	1 (4.76)	0 (0)
8	0 (0–0)	0 (0)	0 (0)	0 (0–0)	0 (0)	0 (0)	0 (0–0)	0 (0)	0 (0)	0 (0–0)	0 (0)	0 (0)
9	0 (0–0)	0 (0)	0 (0)	0 (0–0)	0 (0)	0 (0)	0 (0–0)	0 (0)	0 (0)	0 (0–0)	0 (0)	0 (0)

## Data Availability

All the data used in this study are available from the corresponding author.
